# Effects of xylazine-ketamine anesthesia on plasma levels of cortisol and vital signs during laparotomy in dogs

**Published:** 2014-09-17

**Authors:** H. Naddaf, H. Najafzade Varzi, S. Sabiza, H. Falah

**Affiliations:** 1 *Department of Clinical Sciences, Faculty of Veterinary Medicine, Shahid Chamran University of Ahvaz, Ahvaz, Iran*; 2 *Department of Basic Sciences, Faculty of Veterinary Medicine, Shahid Chamran University of Ahvaz, Ahvaz, Iran*; 3 *Department of Clinical Sciences, Faculty of Medicine, Jondishapour University, Ahvaz, Iran*

**Keywords:** Anesthesia, Cortisol, Dog, Vital signs, Xylazine-ketamine

## Abstract

This study was designed to evaluate effects of xylazine-ketamine anesthesia on plasma levels of cortisol and vital signs during and after laparotomy in dogs. Eight clinically healthy, adult male dogs, weighing 20 kg were used. All dogs were initially sedated by acepromazine. Thirty minutes later, ketamine plus xylazine was used to induce anesthesia. Surgical incision of laparotomy was done. After a 5 min manipulation of the abdominal organs, the incision was sutured. Vital signs including heart rate, respiratory rate and rectal temperature (RT) were recorded at the times of -30: premedication, 0: induction and Surgical incision, 30: End of surgery, 60, 90 and 120 min. Blood was sampled at the above mentioned times and analyzed using a commercial ELISA kit for cortisol. A significant decreasing trend in RT was observed during the studied times. No significant changes were observed in heart rate and respiratory rate (*p*>0.05), except at the time of 60 respiratory rate significantly decreased when compared to the time of 90 (*p*=0.026) and 120 (*p*=0.041). A non-significant but increasing trend in plasma levels of cortisol was observed.

## Introduction

For a long time a search has been made for the utilization of techniques and concepts that will make anesthesia safe and applicable to the diverse surgical procedures in veterinary medicine, resulting in the usage of various anesthetic protocols and associations during routine surgeries (Mathews, 2000; Mattos Junior *et al.*, 2009).

Pain is an unpleasant sensory or emotional experience most commonly associated with actual or potential tissue damage. The sensation of pain is a consequence of the activation of specialized receptors and neurological pathways after such nocuous stimulus. Studies of acute clinical pain have most often evaluated the effects of surgical trauma on animals, while prevention and pain management are the key issues in anesthesia (Tranquilli *et al.*, 2007; Gaynor and Muir, 2008).

Pain evaluation in animals has received an increasing attention over the past decade as the veterinary profession has come to acknowledge that animals are likely to experience and suffer from pain in a similar way to humans. When pain is not appropriately managed, it is not only an animal welfare issue, but it can also have many detrimental effects which can impact the patient recovery (Orskov, 2010). Unfortunately, behavioral expression is highly species specific and is also influenced by age, breed, individual temperament and the presence or absence of additional stressors such as anxiety or fear (Mathews, 2000).

There are also a variety of physiological changes which can occur in response to pain such as increases in heart rate, respiratory rate, blood pressure and body temperature. Obviously, there are a variety of other factors that can affect these physiological parameters such as stress, hypovolemia and intercurrent diseases (Atalan *et al.*, 2002; Gaynor and Muir, 2008; Bergamasco *et al.*, 2010).

The assessment of the neuroendocrine response has been accepted as a reasonable surrogate for surgical stress and pain in animal models and it is found that cortisol is a sensitive blood marker for evaluation of pain (Dart, 1999; Ko *et al.*, 2000; Yoder and Wolf, 2005). Due to common use of anesthesia induced by Xylazine plus ketamine in our clinic, the aim of this study was to evaluate the effects of laparotomy under xylazine plus ketamine-induced anesthesia on the plasma levels of cortisol and vital signs in dogs that underwent laparotomy.

## Materials and Methods

The project was approved by the Institutional Animal Care and Use Committee of Shahid Chamran University of Ahvaz. Eight adult and clinically healthy male dogs of mixed breed with an average weight of 20 kg were used. One month before the operation, vaccination with anti Rabbies (Merial Co, Czech republic) and DHPPi+L (BioVeta Co, Czech republic) vaccine was done, and also Anti-parasitic treatment was given with the routine dose of Ivermectin 5% (Erfan Co, Iran), Mebendazole (Modava Co, Iran) and Peraziquantel (Damloran Co, Iran).

After 12 hours of food restriction, all dogs were initially sedated by acepromazine (0.2 mg/kg, IV, Alfasan Co, Netherlands) (Tranquilli *et al.*, 2007). 30 min later, Ketamine 10% (10 mg kg^-1^, IV, Alfasan Co, Netherlands) plus xylazine 2% (1 mg kg^-1^, IV, Alfasan Co, Netherlands) was used to induce anesthesia (Tranquilli *et al.*, 2007). Alternatively, ketamine 10% (10 mg/kg) was intermittently applied according to the dog’s reactions for maintaining the duration of the anesthesia when the number of respiratory rate and heart rate elevated or the tongue shacked. Then, surgical incision for laparotomy was done (Tranquilli *et al.*, 2007).

During surgery, fluid therapy was given using lactate ringer (10 ml/kg/h) (Tranquilli *et al.*, 2007). A 5 min manipulation of the abdominal organs was performed. In all cases, the manipulation involved only the exposure of the spleen and exposure and pinch of a loop of jejunum. The abdominal incision was sutured using the current method. All surgeries were done during morning and by one specified surgical team so that the duration and quality of all surgeries was approximately the same.

In order to measure plasma levels of cortisol, the blood was sampled at the times of -30: premedication, 0: induction and Surgical incision, 30: End of surgery, 60, 90 and 120 min from the cephalic vein. In this procedure (the time of -30 min was time of sedation and 0 was time of induction).

Samples were collected into heparinized tubes and were immediately centrifuged for 10 min at 2500 rpm. The plasma was kept at -20 °C until the samples were analyzed using a commercial ELISA kit of cortisol (DiaMetra Co. Italy). Vital signs including heart hate (HR), respiratory hate (RR) and rectal temperature (RT) were recorded at the times of -30, 0, 30, 60, 90 and 120 min.

Statistical analysis of the collected data was carried out using IBM SPSS Statistics (Release 19, SPSS Inc., Chicago, Illinois, USA). The residuals were tested for normality by Shapiro-wilk’s test and normality plots (histogram and quantile plots) and for homogenicity of variation by Levene’s test and examining residual plot. Statistical analysis of data was assessed using one-way analysis of variance (ANOVA).

Multiple comparisons were made by using post-hoc tests (Tukey’s method) to find which groups were significantly different from each other. Data present are presented as mean±standard error (mean±SEM). The level of significance was defined as *p*<0.05.

## Results

**Fig. 1:**
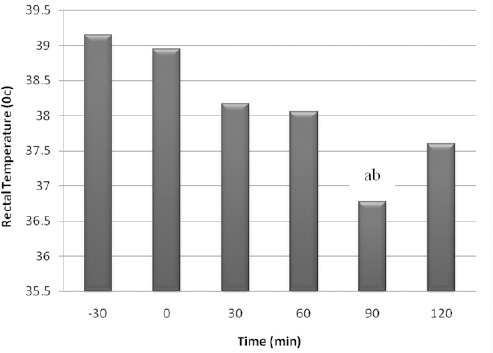
Mean ± SEM of rectal temperature. The letters (a: -30, b: 0, c: 30, d: 60, e: 90, f: 120) show significant difference between times of the study (*p*<0.05, n=8).

A decreasing trend in RT was observed. A decreasing change was observed after the time of 60, so that the minimum temperature was recorded at the time of 90 min. This decrease was significant between the time of 90 and 0 (*p*=0.042) and also between the time of 90 and -30 (*p*=0.004). Also, RT changed significantly between time of 120 and 0 (*p*<0.05). RT changes are shown in Figure 1.

**Fig. 2:**
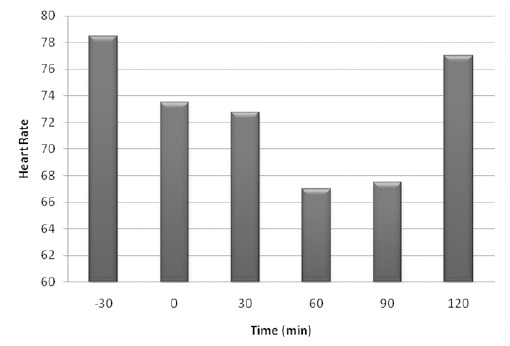
Mean ± SEM of heart rate. No significant changes were observed between times of the study (*p*>0.05, n=8).

**Fig. 3:**
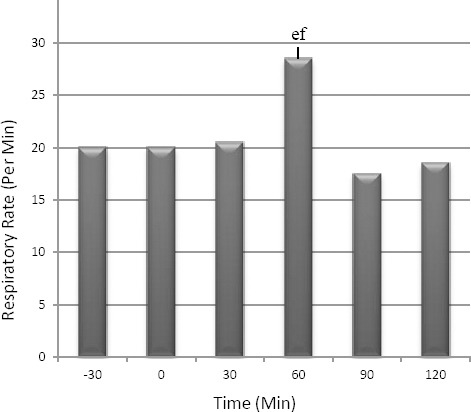
Mean ± SEM of respiratory. The letters (a: -30, b: 0, c: 30, d: 60, e: 90, f: 120) show significant difference between times of the study in the control group (*p*<0.05, n=8).

No significant changes were observed in heart rate and respiratory rate, except at the time of 60 min where respiratory rate significantly decreased by comparison with the time of 90 (*p*=0.026) and 120 min (*p*=0.041). Heart rate and respiratory rate changes are shown in Figures 2 and 3.

**Fig. 4:**
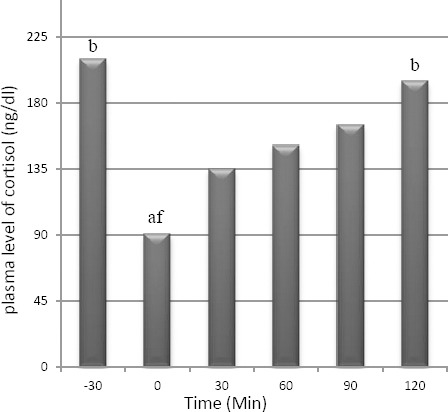
Mean ± SEM of plasma level of cortisol. The letters (a: -30, b: 0, c: 30, d: 60, e: 90, f: 120) show significant difference between times of the study (*p*<0.05, n=8).

The minimum levels of cortisol were recorded at the time of 0 min. Plasma level of cortisol significantly decreased after administration of acepromazine where the difference between 0 min and -30 was significant (*p*<0.05). An increasing trend of cortisol level was observed after induction of anesthesia that was only significant at the time of 120 min (*p*<0.05) compared to time zero. Plasma level of cortisol changes are in Figure 4.

## Discussion

Surgical procedures are associated with a complex stress response characterized by neurohormonal, immunological, and metabolic alterations which are intimately related to the severity of the lesion, duration of surgery, and the clinical status of the patient (Mattos Junior *et al.*, 2009). These responses induce a wide range of alterations in diverse organs and systems, which have resulted in various studies aimed at minimizing or inhibiting these alterations.

In an attempt to identify painful processes in animals, various authors have proposed the evaluation of the levels of serum cortisol and plasma catecholamine levels and thereby identifying techniques and safe anesthesia associations relative to the well-being and ethics of animal handling (Tranquilli *et al.*, 2007; Mattos Junior *et al.*, 2009).

Acute pain is the result of a traumatic, surgical, or infectious event. Acute pain has a biological function in that it serves as a warning that something is wrong. Nociceptive stimulation of surgical site causes hyperventilation, increased hypothalamic neural sympathetic tone, and increased release of catecholamines and other endocrine hormones which consequently increase blood pressure, cardiac work, and myocardial oxygen consumption. In addition, there is an increased secretion of cortisol (Tranquilli *et al.*, 2007; Gaynor and Muir, 2008).

Pain is generally alleviated using analgesic or anesthetic drugs during and post-surgery. It is very important to use an anesthetic procedure to alleviate pain of the patient under surgical operation. Less expensiveness and easy to use, injectable anesthesia is more preferable than inhalation anesthesia for small animal surgeries.

Xylazine plus ketamine is an injectable anesthesia used in small animal surgeries. Xylazine is one of the alpha-2 adrenergic agonists and produces dose-dependent sedation, analgesia and muscle relaxation. However, alpha-2 agonists profoundly alter cardiovascular function by producing bradycardia, hypertension followed by hypotension, decreased myocardial contractility, and dysarhythmias (Dart, 1999; Hall *et al.*, 2001; Tranquilli *et al.*, 2007; Plumb, 2008).

Evaluating plasma level of cortisol in this study showed a significant decline after administration of acepromazine. Acepromazine depresses the CNS and cardiovascular system, modifying the responses to noxious stimuli (Cassu *et al.*, 2008; Tranquilli *et al.*, 2007; Plumb, 2008).

In this study, an increasing trend in plasma level of cortisol was observed after induction of anesthesia but the changes during surgery were not significant; so this anesthesia procedure seems to be suitable for laparotomies. Many studies suggest that cortisol secretion from the adrenal cortex increases rapidly following the start of surgery, as a result of stimulation by ACTH (Shutt *et al.*, 1987; Desborough, 2000; Ko *et al.*, 2000). The cortisol response can be modified by anesthesia intervention (Shutt *et al.*, 1987; Desborough, 2000; Ko *et al.*, 2000).

Monitoring of heart rate and respiratory rate in this study showed that all changes were not significant between times of the study, except at one time of this study (Moens and Fargetton, 1990; Principe, 2003; Picollo *et al.*, 2012). In this study rectal temperature monitoring suggests that it had a partial significant decreasing trend between times of the study. It could be hypothesized this result is a cause of acepromazine injection as suggested by some studies (Moens and Fargetton, 1990; Cassu *et al.*, 2008; Tranquilli *et al.*, 2007; Plumb, 2008; Mattos Junior *et al.*, 2009; Picollo *et al.*, 2012).

The use of serum cortisol has been recognized as one of the most efficient methods to evaluate pain in small animals and humans, and is therefore of importance to evaluate the analgesic efficiency of different drugs by measuring the serum cortisol levels. The type of tissue damage induced influences the metabolic response of the organism. In dogs that were subjected to different surgical procedures and systemic diseases and anesthetized by thiopental and halothane, it was concluded that the act of anesthesia by itself caused an increase in the levels of cortisol; even though all other situations demonstrated increase in plasma cortisol (Mattos Junior *et al.*, 2009).

Similar results were observed when ovariohysterectomy was performed with nociceptive stimulation associated with anesthesia based on protocols with acepromazine, propofol and isoflurane, during which it was suggested that the increase in cortisol was due to only anesthesia, even though statistical differences were not observed (Shutt *et al.*, 1987; Desborough, 2000; Mattos Junior *et al.*, 2009).

The results from this study were similar to those previously described, notwithstanding the anesthetic association; a gradual increase in plasma cortisol was observed (Shutt *et al.*, 1987; Desborough, 2000; Mattos Junior *et al.*, 2009).

Study limitations include the small number of dogs and the pain was not scored. Based on the results obtained from the experiments, we suggest the use of xylazine plus ketamine induced anesthesia for laparotomy in dogs. No significant change in plasma cortisol level and vital signs were observed during and post-surgery in this study.
